# Proximate Analysis of *Moringa oleifera* Leaves and the Antimicrobial Activities of Successive Leaf Ethanolic and Aqueous Extracts Compared with Green Chemically Synthesized Ag-NPs and Crude Aqueous Extract against Some Pathogens

**DOI:** 10.3390/ijms24043529

**Published:** 2023-02-09

**Authors:** Mostafa Ahmed, Diaa Attia Marrez, Nadia Mohamed Abdelmoeen, Ebtesam Abdelmoneem Mahmoud, Mohamed Abdel-Shakur Ali, Kincső Decsi, Zoltán Tóth

**Affiliations:** 1 Festetics Doctoral School, Institute of Agronomy, Georgikon Campus, Hungarian University of Agriculture and Life Sciences, 8360 Keszthely, Hungary; 2 Department of Agricultural Biochemistry, Faculty of Agriculture, Cairo University, Giza 12613, Egypt; 3 Food Toxicology and Contaminants Department, National Research Centre, Dokki, Cairo 12622, Egypt; 4 Institute of Agronomy, Georgikon Campus, Hungarian University of Agriculture and Life Sciences, 8360 Keszthely, Hungary

**Keywords:** *Moringa oleifera*, pathogenic bacteria, plant infecting fungi, Ag-NPs, chemical composition

## Abstract

Research on the use of different parts of the *Moringa oleifera* plant as a nutritional and pharmaceutical resource for human and animals has increased in recent years. This study aimed to investigate the chemical composition and the TPCs and TFCs of Moringa leaves, the antimicrobial activities of Moringa successive ethanolic, aqueous, crude aqueous extracts, and green-chemically synthesized characterized Ag-NPs. The results indicated that the ethanolic extract recorded the highest activity against *E. coli*. On the other side, the aqueous extract showed higher activity, and its effects ranged from 0.03 to 0.33 mg/mL against different strains. The MIC values of Moringa Ag-NPs against different pathogenic bacteria ranged from 0.05 mg/mL to 0.13 mg/mL, and the activity of the crude aqueous extract ranged from 0.15 to 0.83 mg/mL. For the antifungal activity, the ethanolic extract recorded the highest activity at 0.04 mg/mL, and the lowest activity was recorded at 0.42 mg/mL. However, the aqueous extract showed effects ranging from 0.42 to 1.17 mg/mL. Moringa Ag-NPs showed higher activity against the different fungal strains than the crude aqueous extract, and they ranged from 0.25 to 0.83 mg/mL. The MIC values of the Moringa crude aqueous extract ranged from 0.74 to 3.33 mg/mL. Moringa Ag-NPs and their crude aqueous extract may be utilized to boost antimicrobial attributes.

## 1. Introduction

Because of their high nutritional content and low anti-nutritional components, *Moringa oleifera* products have gained prominence and are used for livestock and human diets. Recent studies of the nutrients found in the plant’s leaves, seeds, and stems have revealed that they are high in protein, vital amino acids, minerals, and vitamins such as vitamin C, which prevents scurvy, as well as some B-Complexes and other bioactive chemicals [1,2]. Additionally, because they are low in calories, Moringa leaves can be included in an obese person’s diet. Fibrous pods are useful for treating digestive issues and preventing colon cancer [3,4]. According to the research, immature pods have a fiber level of about 46.78 percent and a protein content of about 20.66%. The immature pods and flowers showed similar quantities of palmitic, linolenic, linoleic, and oleic acids, whereas the leaves and flowers had higher amino acid contents than the pods (30%, 44%, and 31%). Among the several elements found in Moringa that are necessary for growth and development, calcium is regarded as one of the key minerals for human growth [5]. The significant variance in the nutritional composition may be caused by elements including the growth environment, harvesting stage, type of soil, and processing technique. Significant levels of vital minerals, vitamins, amino acids, and fatty acids are present in the leaves and seeds [6,7].

*M. oleifera* is significant for its therapeutic effects [8] as well as its high nutritional content. As cardiac and circulatory stimulants, this plant’s leaves, roots, seeds, bark, fruits, flowers, and immature pods also have antioxidant, diuretic, antihypertensive, cholesterol-lowering, antiulcer, and antispasmodic properties according to [9,10]; antipyretic, antiepileptic, anti-inflammatory, antifungal, and antibacterial properties according to [10,11]; and antitumor properties according to [9,12]. Many in vitro studies have demonstrated the inhibitory activity of the variant extracts from different parts of *M. oleifera* on Gram-positive bacteria (*Enterococcus faecalis*, methicillin-resistant *Staphylococcus aureus*, and *Staphylococcus epidermidis*) and Gram-negative bacteria (*Salmonella enterica*, *Pseudomonas aeruginosa*, *Klebsiella pneumoniae*, and *Escherichia coli*) isolated from hospital samples [13,14]. The antimicrobial activity of the crude extracts on *E. coli* and *K. pneumoniae* strains have been compared with that of the antibiotic streptomycin [15,16].

Understanding the molecular makeup of the target species that will be used as a source of biomolecules should be the first step in the logical planning of a green synthesis approach toward the creation of silver nanoparticles (Ag-NPs) [17]. In fact, when choosing a suitable biological resource, the balance between the profiles of the molecular components should be one of the most important factors to take into account; otherwise, it will be an entirely empirical trial-and-error method. Unexpectedly, only a small number of research studies have assessed or at least published the bioresource substances used to synthesize Ag-NPs. Additionally, given the current “omics” era, this is an unexpected finding given that there are molecular profiles from multiple potential studies involving creatures from all Earth biomes [18].

The current research aimed to investigate the chemical composition of Moringa leaves, the polyphenolic burden in variant successive extracts of Moringa leaves, the antimicrobial activity of the ethanolic and aqueous successive extracts, and to study the effect of green chemically synthesized characterized Ag-NPs as antimicrobial agents compared with leaf ethanolic and aqueous successive and crude aqueous extracts.

## 2. Results

### 2.1. Proximate Analysis and Mineral Content of Moringa Leaves

Moringa leaves were analyzed for moisture content (DM% and OM%), total lipids, nitrogen-free extract (NFE%), total protein (TP%), ash, and crude fiber (CF%). The results are demonstrated in Table 1. The mineral content of dried Moringa leaves is shown in Table 2. The elements’ concentrations were 1110 ± 7.00 ppm for Ca, 21.50 ± 1.50 ppm for Na, 147.5 ± 10.5 ppm for Mg, 559.00 ± 14.05 ppm for K, 0.125 ± 0.05 ppm for Zn, and 0.53 ± 0.09 ppm for Cu. It can be observed that Moringa leaves are rich in Ca, K, Mg, and Na.

### 2.2. Polyphenolic Content in Moringa Leaves’ Successive Extracts

The total phenol content (µg/g GAE) in Moringa—expressed in GAE—was varied between 12.72 ± 0.13 µg/g in petroleum ether extract and 32.96 ± 0.58 µg/g in ethyl acetate extract, as well as 32.66 ± 0.21 µg/g in aqueous extract (Table 3). The total flavonoid content (µg/g QE) in Moringa—expressed in quercetin equivalents—was varied between 117.40 ± 5.03 µg/g in petroleum ether extract and 804.73 ± 12.02 µg/g in aqueous extract (Table 3). Significantly, the highest results were found in aqueous extract followed by ethanolic and ethyl acetate extracts with 540.07 ± 12.98 and 462.73 ± 1.76 µg/g, respectively.

### 2.3. Antimicrobial Activity of Moringa Leaves' Successive Ethanolic and Aqueous Extracts

It was reported that the aqueous extract had the highest antibacterial activity against *B. cereus*, *E. coli*, and *P. aeruginosa* with an inhibition zone of 7.66 ± 0.16, 10.00 ± 0.29, and 10.67 ± 0.60 mm, respectively (Table 4). The aqueous extract showed the most powerful ability to inhibit microbial growth against the following different phytopathogenic fungal strains: *A. flavus*, *A. parasiticus*, *A. niger*, *A. carbonarius*, *A. ochraceus*, *A. westerdijikia*, *F. proleferatum*, and *P. verrucosum*, with an inhibition zone of 8.50 ± 0.76, 10.33 ± 0.60, 12.67 ± 0.73, 10.50 ± 0.29, 10.83 ± 0.93, 9.50 ± 0.29, 10.00 ± 0.2, and 11.00 ± 1.04 mm, respectively (Table 5).

Ethanolic extract recorded the highest activity against *E. coli* with 0.18 ± 0.07 mg/mL, followed by 0.42 ± 0.08 mg/mL with *Salmonella typhi*, and the lowest activity was recorded against *Staph. sciuri* with 4.17 ± 0.83 mg/mL. Aqueous extract showed high activity, and its effects ranged from 0.03 ± 0.01 mg/mL to 0.33 ± 0.08 mg/mL. There was no significant difference between diverse concentrations against all foodborne pathogenic bacteria (Table 6). Ethanolic extract recorded the highest activity against *A. parasiticus* and *A. flavus* with 0.04 ± 0.01 mg/mL, and the lowest activity was recorded against *F. Proliferatum* and *A. carbonarius* with 0.42 ± 0.08 mg/mL. Aqueous extract showed effects ranging from 0.42 ± 0.08 mg/mL to 1.17 ± 0.33 mg/mL, and there was no significant difference between diverse concentrations against all phytopathogenic fungi (Table 7).

### 2.4. Antimicrobial Activity of Moringa Crude Aqueous Extracts and Synthesized Ag-NPs

The crude leaf aqueous extract from Moringa showed a different ability to inhibit the growth of the foodborne pathogenic bacterial and fungal strains as shown in Table 8. The crude leaf aqueous extract from Moringa had the highest antibacterial activity against *P. aeruginosa* with an inhibition zone of 8.83 ± 0.44 mm, followed by *S. aureus* and *S. enterica* with inhibition zones 8.17 ± 0.17 and 8.00 ± 0.29 mm, respectively. Ag-NPs from Moringa showed higher ability than the crude aqueous extract to inhibit the growth of the different foodborne pathogenic bacteria, and there was no significant difference between diverse strains in their responses to the synthesized Ag-NPs from Moringa, as the maximum inhibition was against *P. aeruginosa* with inhibition zone 10.67 ± 0.17 mm and the minimum one was against *B. cereus* with inhibition zone 9.17 ± 0.44 mm.

There was no significant difference between diverse phytopathogenic fungal strains in their responses to the crude leaf aqueous extract from Moringa, as the maximum inhibition was against *A. flavus* and *A. westerdijikia* with the same inhibition zone of 8.33 ± 0.60 mm, and the minimum one was against *A. parasiticus*, *A. carbonarius*, and *A. ochraceus* with the same inhibition zone of 7.83 ± 0.17 mm. Ag-NPs from Moringa also showed no significant inhibition ability against the different phytopathogenic fungi; the different strains were inhibited in close values, as the maximum inhibition zone was against *Penicillium verrucosum* with 10.33 ± 0.44 mm and the minimum one was against *A. parasiticus* with 9.00 ± 0.00 mm (Table 9).

Moringa Ag-NPs showed MIC values against different foodborne pathogenic bacteria, and their effects ranged from 0.05 ± 0.00 mg/mL to 0.13 ± 0.06 mg/mL. The activity of crude aqueous extract from Moringa also ranged from 0.15 ± 0.05 mg/mL to 0.83 ± 0.08 mg/mL (Table 10). Moringa Ag-NPs showed activity against the different strains of the phytopathogenic fungi, ranging from 0.25 ± 0.00 mg/mL to 0.83 ± 0.17 mg/mL. The MIC values of Moringa crude leaf aqueous extract ranged from 0.74 ± 0.14 mg/mL to 3.33 ± 0.83 mg/mL (Table 11).

## 3. Discussion

The moisture percent was determined to be 7.94 ± 0.10%, DM (dry matter) was 92.06 ± 0.10%, and OM (organic matter) was 88.35 ± 0.54%. The value of organic matter obtained in this study was slightly lower than the value (93.7%) obtained by [19]. The nitrogen-free extract (sugars and starches) was determined to be 14.05 ± 2.68% (dry weight), and this was much lower than the total carbohydrates (63.11 ± 0.09%) determined by [19]. The total lipids were determined to be 13.55 ± 1.10%. The total protein value reported by [20] was lower (27.44%) than the value obtained in this study (28.59 ± 0.11%) (dry weight), but Mutayoba et al. (2011) reported much higher (30.65%) crude protein in Moringa leaves. The ash was determined to be 11.65 ± 0.54%, and the crude fiber was 32.15 ± 1.87%. These findings were much higher than those reported by [21].

Due to its high nutritional content and beneficial physiological characteristics, Moringa is regarded as a good food source [22]. Water, protein, sugar, mineral salts, and fatty acids are the main components of MO leaves [22]. L-arabinose, D-mannose, D-galactose, L-rhamnose, and D-xylose are a few of these sugar compounds that are pharmacologically active and have been demonstrated in numerous large-scale investigations to improve wound healing. Furthermore, numerous studies have shown that natural sugars with diverse antibacterial properties include D-mannose and D-glucose. Additionally, Moringa leaves contain a variety of fatty acids with molecular structures similar to 10-HDA, such as lauric acid, myristic acid, palmitic acid, arachidonic acid, and oleic acid [23]. In numerous in-depth investigations, the bioactive substance 10-HDA, which is present in royal honey, has been demonstrated to improve wound healing. The transforming growth factor-1 (TGF-1) and vascular endothelial growth factor (VEGF) are two growth factors that these type of fatty acids causes fibroblasts to produce in wounds [24].

One of the most significant subgroups of the secondary metabolites produced by plants is the phenolic chemicals. In order to scavenge free radicals, phenolics with at least one aromatic ring (C6) and one or more hydroxyl groups are ideal candidates [25]. Generally, phenolic chemicals act as potential metal chelators and prevent lipid peroxidation by quenching free radicals and producing phenoxy radicals that are stabilized by resonance. The most important family of natural phenolics and flavonoids can directly scavenge re-oxygenic species and has the capacity to provide electrons or hydrogen atoms in a timely manner [26]. This led to the measurement of the total phenol content (TPC) and total flavonoid content (TFC) of Moringa.

It was noticed that ethyl acetate, aqueous and ethanolic extracts were richer in total phenol content than other extracts, which suggested the presence of water-loving (polar) phenolic compounds. Additionally, total phenol content was relatively higher in hexane extract than in petroleum ether extract, which indicated that the highest amount of present non-polar phenolic compounds was extracted in hexane yield. These results were well-correlated with previous studies [27]. They reported that the higher recovery of phenolic compounds was in polar solvents’ yields followed by non-polar ones. Adebayo et al. (2018) determined the phenolic and flavonoid contents in Moringa leaves to be varied from 13.61 to 20.42 mg gallic acid equivalence/g sample and 0.58 to 9.81 mg quercetin equivalence/g sample, respectively [28]. Al-Owaisi et al. (2014) reported that *M. oleifera* leaves exhibited the richest phenolic compounds and flavonoid contents among different species of Moringa [29]. Ethanolic extract of *M. oleifera* leaves showed higher phenolic compounds and flavonoid content than that reported by [30]. In the previous study by Adebayo et al. (2018), *M. oleifera* leaves showed higher phenolic content than what was reported by [31], who used 80% ethanolic extract and obtained 8.21 µg/100 g, while in contrast, the results of this study were less than theirs in the flavonoid content with the same solvent (531.2 mg/g). That study on *M. oleifera* leaves was supported by [32] regarding the ethanolic extract in phenolic and flavonoid content determination and the moderate action of ethyl acetate extract, but also contradicted them for the hexane extract since they did not exhibit any phenolic compounds or flavonoids.

The aim of experimenting with different Moringa successive extracts of different and varying degrees of polarity to determine the phenolic content was to investigate the successive extracts which contain huge amounts of C6 compounds. In particular, the high phenolics and flavonoid content of ethanolic and aqueous successive extracts in Moringa was the critical dependent point of that research. After the determination of TPCs and TFCs in the different successive extracts from Moringa leaves, it was clear that the successive ethanolic and aqueous extracts are the most promising successive extracts to be tested as antimicrobials against some foodborne pathogenic bacteria and some of the other phytopathogens. It was expected that the positive effects of these polar extracts as antimicrobials will be significant against the largest number of microbial strains. Besides investigating Moringa successive extracts to detect which of them contains a huge amount of phenols, then to detect their effects as promising antimicrobials, there was a crucial purpose to reach the commercial and eco-friendly successive extract, then to use it in the creation of silver nanoparticles at the cheapest cost. It was clear that the extract that fits these criteria is the aqueous extract. Therefore, the aqueous extract was the template that was used to synthesize the silver nanoparticles in a newly developed technique.

In order to combat the many types of pathogenic bacteria that are resistant to or less sensitive to the present and traditionally known antibiotics, there is an alarming rise in the number of bacterial strains that are resistant to a variety of antimicrobials [33,34]. Scientific research into the antimicrobial properties of many plants has revealed that a wide variety of plant products can impede the growth of dangerous bacteria. Many of these drugs looked to have unique structures and modes of action from those of the antibiotics now in use [35]. The leaves of *M. oleifera* contain numerous phytochemicals with antimicrobial properties, including tannins, phenolic compounds, and flavonoids [36,37]. As a result, it would suggest that the antifungal activity observed in these research studies could be attributed to such compounds. Leaf extracts of *M. oleifera* have been found to be effective in controlling the growth of fungi such as *Basidiobolus haptosporus* and *Basidiobolus ranarums* [38]. Devendra et al. (2011) reported that chloroform (semi-miscible solvent) extract from Moringa leaves showed a growth inhibition against some pathogenic bacteria, such as *Escherichia coli* (MTCC 443), *Pseudomonas aeruginosa*, *Staphylococcus aureus* (MTCC 3160), and *Streptococcus pyogenes* (MTCC 442), and also with some of the phytopathogenic fungi, such as *Aspergillus niger* (MTCC 1781) and *Candida albicans* (MTCC 181), as compared with petroleum ether (completely immiscible “non-polar” solvent) extract, which was not able to inhibit bacterial and fungal strains [39]. Aqueous and ethanolic extracts of *M. oleifera* leaves have shown antibacterial activity by producing zones of inhibition using the disc diffusion method against *Escherichia coli*, *Proteus vulgaris*, and *Salmonella typhi*, respectively, but acetone and chloroform extracts with non-polar natures did not possess any antibacterial activity [40].

Broad-spectrum activity against all the investigated species of foodborne pathogenic bacteria has been reported for Moringa leaf aqueous extract. According to Patel (2001), Moringa leaves are frequently used in the treatment of bacterial infection, fungal infection, and diarrhea [41]. This is because Moringa leaves contain chemical compounds such as kaempferol and rutin, which are said to have antibiotic and antioxidant properties that are linked to the inhibition of microorganisms. When compared with *Staph. sciuri*, *P. aeruginosa*, and *Staph. aureus*, the ethanolic extract of Moringa leaves showed higher inhibitory characteristics against *E. coli*, *S. enterica*, *S. typhi*, and *B. cereus*. These results were in agreement with a prior study [42].

FTIR studies of Moringa aqueous extracts and Ag-NPs synthesized by Moringa crude aqueous extract and NaBH_4_ were performed to characterize the chemical structure of the nanoparticles. The FTIR spectrum of Ag-NPs synthesized by Moringa leaves showed the presence of different peaks which were not detected in the FTIR spectra of Moringa crude aqueous extract or which have been shifted. A strong peak at 3409.53 cm^−1^ corresponds to the combined peaks of OH group stretching vibration in carboxylic acids. The bands at 2917.77 cm^−1^ and 2948.31 cm^−1^ in the FTIR spectrum indicated C-H stretching of the alkane amide I band of proteins, and these results were matched with Ganapathy and Sivakumar (2015) [43]. A band at 2364.3 cm^−1^ indicated alkynes. The band at 1631.48 cm^−1^ corresponded to amine groups of N–H bending vibrations of proteins and was characteristic of C=O carbonyl groups [34]. The bands at 1463.71 cm^−1^ and 1425.14 cm^−1^ indicated nitro group N=O. The peaks at 1240 cm^−1^, 1078.98 cm^−1^, and 1029.8 cm^−1^ corresponded to C-O stretching from alcohol, ether, ester, and carboxylic acid. The peaks at 915.058 cm^−1^, 828.277 cm^−1^, 828.277 cm^−1^, 759.816 cm^−1^, 595.896 cm^1^, and 474.404 cm^−1^ indicated a fingerprint region. They could also indicate C-N imines. The leaves of Moringa may contain high amounts of reductones (enediols with a carbonyl group adjacent to the enediol group), and the highly reducing action of Ag-NPs compared with the crude extracts could be credited to the presence of phenolic functional groups on the surface of synthesized Ag-NPs [44], which enable them to be more effective as antimicrobials [45] and antioxidants [46].

Peixoto et al. (2011) found that aqueous ethanolic extracts of Moringa leaves showed effective antibacterial activity against *Staphylococcus aureus*, *Vibrio parahaemolyticus*, *Enterococcus faecalis*, and *Aeromonas caviae*, whereas no effects were seen against *Escherichia coli*, *Pseudomonas aeruginosa*, and *Salmonella enteritidis* [16]. Out of the 19 Gram-negative bacteria strains tested, the trials by [47] demonstrated that the methanolic extract of Moringa leaves constrained 13 different bacterial strains [47]. These strains included *Escherichia coli*, *Klebsiella pneumonia*, and *Pseudomonas aeruginosa*. Moringa leaf extract with antibacterial properties contain alkaloids, polyphenols, flavonoids, anthraquinones, coumarins, tannins, triterpenes, sterols, saponins, and certain secondary metabolites, according to further proximate analysis. It was proposed that Moringa leaf extracts could be used alone or in combination with other medicines to treat a variety of infectious disorders.

These previous results support the results of the current study, as the polar extracts from Moringa usually contain the most effective compounds that are mainly responsible for the inhibition and death of different types of pathogenic bacteria. These bactericidal behaviors may be related to some of the internal compounds such as 4-(α-L-rhamnopyranosyloxy) benzyl isothiocyanate, methyl *N*-4-(α-L-rhamnopyranosyloxy) benzyl carbamate, and 4-(β-D-glucopyranosyl-1→4-α-L-rhamnopyranosyloxy)-benzyl thiocarboxamide that were identified from the extract of Moringa [47].

Along with the different chemical components that have antibacterial activity, Moringa also contains a number of bioactive peptides that have been isolated and studied. As an insecticidal compound against *Aedes ageypti*, a lectin from Moringa was first obtained from the seed aqueous extract using chitin column chromatography [48]. *Staphylococcus aureus* growth in contaminated waterways was reduced by the water-soluble lectin that was extracted from Moringa, demonstrating the effectiveness of the lectin’s antibacterial properties [49]. According to Moura et al. (2015), the water-soluble lectin inhibited the growth, survival, and cell permeability of *Bacillus cereus*, *Bacillus pumillus*, *Bacillus megaterium*, *Micrococcus* sp., *Pseudomonas* sp., *Pseudomonas fluorescens*, *Pseudomonas stutzeri*, and Serratia. Besides, it was noticed that after lectin treatment, some of the bacteria exhibited a tendency for biocorrosion [50].

Numerous research claimed that Moringa has the potential to control fungal infestation in plants because phytopathogenic fungi can spread disease and endanger the growth of economically important crops. From the Moringa, a thermostable protein known as Moringa chitin-binding protein (Mo-CBP3) was identified using affinity chromatography and ion exchange chromatography. The in vitro antifungal activity showed by Moringa extracts against different fungal strains in the current study may be attributed to Mo-CBP3; as earlier, it showed promising activity against the phytopathogenic fungi *Fusarium solani*, *Fusarium oxysporum*, and *Colletotrichum gloesporioides* at a very low concentration (0.05 mg/mL) with 62% inhibition within 49 h [51]. Additional research showed that *Fusarium solani*’s mycelia development and conidial vitality were inhibited by Mo-CBP3. It was proposed that Mo-CBP3 triggered cell death and reactive oxygen substances (ROS) formation by interacting with the cell membrane in the fungi [51]. The creation of genetically engineered crops is now considered Mo-CBP3 as a potential antifungal medication because of its physiochemical properties, broad antifungal coverage, and poor toxicity [52].

Silver nanoparticles were demonstrated to be involved in antibacterial action in addition to phytochemical compounds and bacteriocins (antimicrobial peptides). The extract of Moringa leaves, as demonstrated [53], produced silver nanoparticles extracellularly by means of the quick reduction of silver ions to form the crystals of nano silvers. *K. pneumoniae*, *P. aeruginosa*, and *Staph. aureus* bacteria’s development was suppressed by solutions of Ag-NPs from Moringa leaf preparations at a concentration of 25 g/mL [54]. Additionally, they stated that the antifungal effectiveness of the silver nanoparticles made by Moringa was tested using three Candida reference strains. All fungal strains had their growth stopped at a concentration of 6.25 g/mL Ag-NPs, but not with the crude leaf extracts. According to Ankanna et al. (2010), compared with Gram-negative bacteria, Gram-positive bacteria were less resistant to the antibacterial effects of synthesized Ag-NPs from Moringa [55]. This was explained by the peptidoglycan layer’s negative charge, which prevented Ag^+^ ions from freely entering the cell wall and was consistent with the results of this investigation.

Examining the outcomes of antibacterial and antifungal properties for synthesized Ag-NPs and crude aqueous extracts from Moringa, it is believed that the antibacterial activity of crude aqueous extract from Moringa against various Gram-positive and -negative foodborne pathogenic bacteria was caused by affecting the growth and cell permeability of these bacteria. This is because the activity shown by the crude extract may be somewhat related to differences found in the composition of the different types of bacteria. On the other hand, mycelia growth inhibition may be what causes the crude extract’s antifungal efficacy. Some reactive oxygen compounds were thought to originate as a result of interactions with the cell membrane [51]. Additionally, Moringa Ag-NPs caused the bacterial cells to produce silver ions, which increased their antimicrobial properties. Ag-NP activity may be related to Ag-NP preferential attack on the electron transport chain, cell proliferation, and ultimately cell death.

## 4. Materials and Methods

### 4.1. Collecting and Processing of Plant Samples

Moringa was collected from the Egyptian Moringa Association at the National Research Centre (NRC), Dokki, Egypt. Collected plants were identified by the National Research Centre (NRC) Herbarium. Leaves were washed three times using distilled water and the excess water was dried. The plant specimens were then weighed and dried in the shade in a room with active ventilation and perimeter for two weeks. The dried samples were weighed and powdered using a mill. Finely ground samples were kept at −20 °C in dark sealed containers until further use.

### 4.2. Extraction

#### 4.2.1. Successive Extraction

The powdered plant (100 g) from leaves of Moringa was obtained by successive extraction using several solvents gradient in polarity (Hexane, Petroleum ether, Ethyl acetate, absolute ethanol, and double distilled water) with continuous mixing in a shaker. All extracts—except the aqueous extract—were filtered using Whatman No. 1 filter paper. Extracts (filtrate) were concentrated at 40 °C under reduced pressure using a rotary evaporator and then kept in a glass flask. The semi-solid ethanolic and aqueous extracts (residue) obtained were stored in a refrigerator for further use. The aqueous extract was filtered using a Buchner funnel, centrifuged, then applied in a freeze dryer to concentrate it [56].

#### 4.2.2. Crude Aqueous Extraction

The powdered plant (10 g) from leaves of Moringa was obtained by crude aqueous extraction using de-ionized water. The aqueous extract was filtered using a Buchner funnel, centrifuged at 6000 rpm/min, then applied in a freeze dryer to concentrate it. The obtained semi-solid extracts (residue) were kept in a freezer at −20 °C until further use [16].

### 4.3. Chemical Composition of Moringa Leaves

#### 4.3.1. Determination of Moisture

The moisture content in Moringa leaves was determined at 105 °C in an oven [57].

#### 4.3.2. Determination of Total Ash

Total ash (%) in dried Moringa leaves was determined [57]. A well-known weight sample (about 3 g) was dried in an oven (105 °C). The dried material was ashed in a muffle furnace at 450–500 °C until completely combusting (ash turned white/gray or slightly colored). The sample was cooled to room temperature, then weighed. Ash % was calculated as follows:

Ash % = (Weight of ash/Weight of sample) × 100

#### 4.3.3. Determination of Minerals

The minerals potassium (K), sodium (Na), calcium (Ca), magnesium (Mg), zinc (Zn), and copper (Cu) were determined [57]. The sample was ashed as described before. The obtained ash was dissolved using 1 mL HCl conc. at crucible walls. Dissolved samples were transferred to a 50 mL volumetric flask and de-ionized water was added to complete the total volume. The solution was filtered through ash-less filter paper Whatman No. 42 and stored in a refrigerator until a determination by Atomic Absorption Units (GBC 932 AA).

#### 4.3.4. Determination of Crude Fiber

The crude fiber content of each sample was determined by digesting the sample in 1.25% H_2_SO_4_ followed by 1.25% NaOH solution [57].

#### 4.3.5. Determination of Crude Protein Content (Nitrogenous Compounds Content)

Total protein (nitrogenous compounds content) was determined by the conventional micro-Kjeldahl method [57]. A sample of 0.3 g powder of Moringa leaves was weighed into a digestion flask. Ten mL sulfuric acid and 0.5 g digestion mixture (1:4 K_2_SO_4_:CuSO_4_) were added. The sample was then incinerated, the digested sample was quantitatively transferred into the Markham micro-Kjeldahl with the least amount of ammonia-free distilled water, and then 20 mL of 50% NaOH solution was added. A strong current of steam was then passed and the ammonia was distilled into 10 mL of 2.5% boric acid. The ammonia was then titrated against HCl using a mixed indicator (0.4 methylene blue and 0.1 methyl red) until a faint red endpoint was obtained. After correction for the reagent blanks, the titration figures were converted into percentage protein using the following equation:

$$Total\;crude\;protein\%=\frac{Read\times N\;of\;HCl\times 0.014\times Conversion\;Factor\times 100}{Sample\;weight}$$

The conversion factor for Moringa = 5.25.

#### 4.3.6. Determination of Total Lipids

Total lipids were extracted from dried Moringa leaves [57] using the Soxhlet apparatus. Fifty grams of Moringa leaves was added into the extraction thimble and defatted with n-hexane at a siphon rate of 4–5 cycles h^−1^ for 12–15 cycles. Hexane was evaporated using a rotary evaporator, and the percentage of total lipids was calculated as expressed in the following equation:

Total lipids % = (Weight of lipids/Weight of sample) × 100

#### 4.3.7. Determination of Nitrogen-Free Extract

Nitrogen-free extract in Moringa leaves was determined by difference using the following equation:

NFE % = 100% − (Total lipids % + Crude protein % + Ash % + Crude fiber %)

### 4.4. Polyphenolic Burden of Moringa Leaves

#### 4.4.1. Determination of the Total Phenolic Content (TPC)

Total phenolic content was determined using the Folin–Ciocalteu reagent with slight modifications [58]. Initially, 4 mg of plant extracts was weighed, the total volume was completed to 2 mL with double distilled water, and many stocks of the plant extracts were prepared, each one of them having 2000 ppm concentration. A total of 500 μL of extracts was added to 500 μL of Folin–Ciocalteu reagent (1 N), then 2 mL of 20% sodium carbonate was added to the mixture and incubated in the dark at room temperature for 90 min. All determinations were performed in triplicates. The absorbance of the blue color developed was measured at 650 nm using a spectrophotometer (UV mini-1240, UV–VIS spectrophotometer, SHIMADZU) against the reagent blank. Gallic acid was used as a standard in a concentration between 10:100 ppm. The total phenolic content was expressed in μg of gallic acid equivalent per gram of dry extract (μg of GAE/g of extract).

#### 4.4.2. Determination of the Total Flavonoid Content (TFC)

Flavonoid contents were determined in each plant extract according to the aluminum chloride colorimetric method with slight modification [58]. Initially, 4 mg of plant extracts was weighed, the total volume was completed to 2 mL with double distilled water, and many stocks of the plant extracts were prepared, each one of them having 2000 ppm concentration. A total of 500 μL of extracts was added into a series of test tubes, and the analysis was performed in triplicates, then 500 μL of distilled water was added into each test tube. All the test tubes were vortexed well, 300 μL of 5% sodium nitrite was added into all the test tubes, then all the test tubes were vortexed again and allowed to stand for 5 min at room temperature. In total, 300 μL of 10% aluminum chloride was added into all the test tubes, they were well-vortexed again, and allowed to stand for 5 min at room temperature. A total of 2 mL of 1 M sodium hydroxide was added to all the test tubes, they were vortexed well, and left to be incubated in the dark at room temperature for 15 min. Finally, the absorbance of the developed pink color against the reagent blank was measured at 510 nm using a spectrophotometer (UV mini-1240, UV–VIS spectrophotometer, SHIMADZU). Quercetin was used as a standard in a concentration between 100:1000 ppm. The total flavonoid content was expressed in μg of quercetin equivalent per gram of dry extract (μg of QE/g of extract).

### 4.5. Under Heating Crude Aqueous Extraction and Green Chemical Synthesis and Characterization of Ag-NPs from Moringa

Moringa crude aqueous extraction and green chemical synthesis of Ag-NPs were applied according to our previous study [59].

#### 4.5.1. UV–VIS Spectroscopy

The optical properties of Ag-NPs were monitored by UV–VIS absorption spectroscopy (PG Instruments Ltd. T80 UV–VIS spectrophotometer, Loughborough, UK) in the central lab of the Biochemistry Department, Faculty of Agriculture, Cairo University. UV–VIS spectra were recorded in the wavelength range of 300–700 nm.

**Figure 1:**
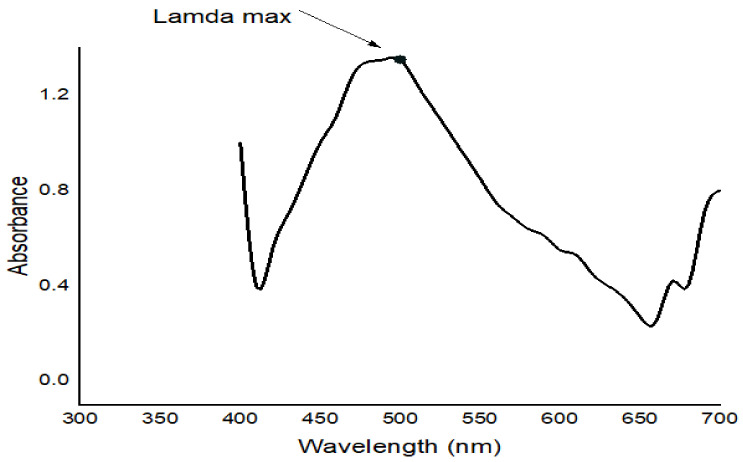
UV–VIS spectrum of Ag-NPs synthesized by the crude aqueous extract from Moringa and NaBH_4_.

The UV–VIS spectrum of Ag-NPs in Figure 1 was adjusted from the reaction medium as an incubation time function. Ag-NPs from Moringa gave absorption peaks between 300 and 700 nm. With time, the absorption intensity increased, indicating an increase in the quantity of formed Ag-NPs. The characteristic feature of the peak at 400–500 nm, which was checked in the UV–VIS spectrum, validated the synthesis of Ag-NPs. This single peak of the plasmon surface resonance indicated that the Ag-NPs were spheres with a wide distribution of size. The UV–VIS spectrum of synthesized Ag-NPs gave absorbance at 472 nm after 24 h of incubation in the dark.

#### 4.5.2. Transmission Electron Microscopy

**Figure 2:**
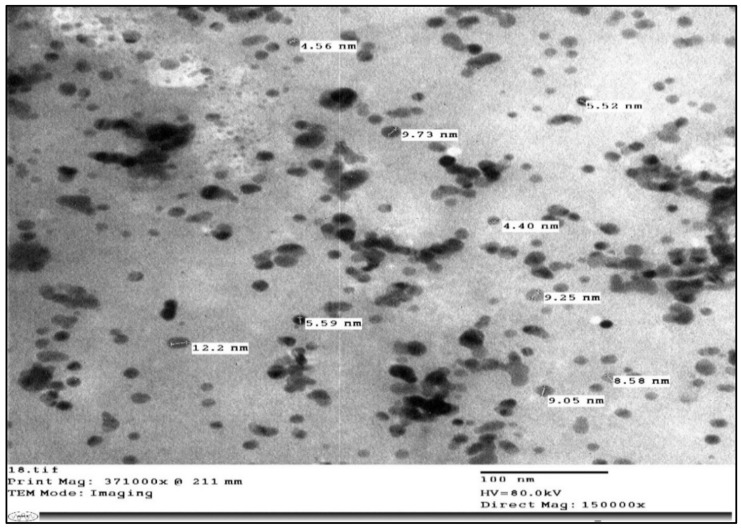
Electron micrographs of silver nanoparticles synthesized by the crude aqueous extract from Moringa and NaBH_4_.

A transmission electron microscope (JEM-1400, JEOL model) was used to determine the morphological screening of Ag-NPs. Imaging was recorded by the electronic microscope lab of Cairo University Research Park (CURP). A glow-discharged grid of carbon was used to place freshly prepared Ag-NPs on and left to be air-dried for a few minutes. Then, the shape and surface harshness of the nanocomposite specimens were checked by a TEM-operated system. TEM of the synthesized Ag-NPs by the crude aqueous extract from Moringa and NaBH_4_ are shown in Figure 2. They ranged from 4.40 to 12.2 nm in size and they were spherical.

#### 4.5.3. Fourier Transform Infrared Spectroscopy (FTIR)

**Figure 3:**
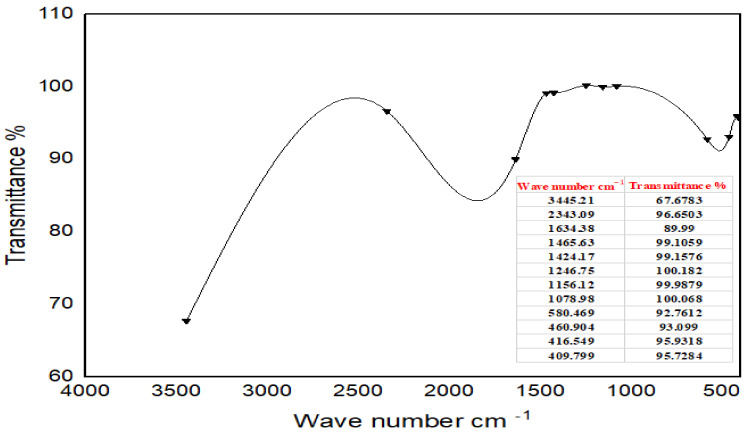
FTIR spectra of Moringa crude aqueous extract.

**Figure 4:**
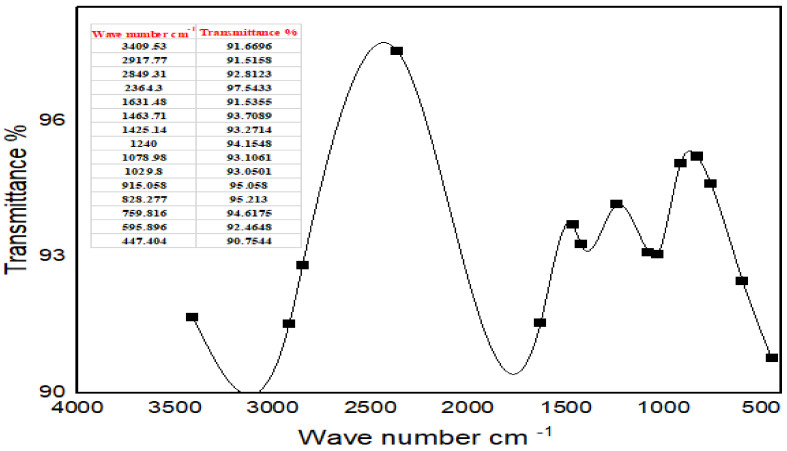
FTIR spectra of silver nanoparticles synthesized by the crude aqueous extract from Moringa and NaBH_4_.

The freeze-dried crude aqueous extracts of Moringa and also the Ag-NPs powders were prepared in the central lab of the Nawah foundation. The samples were prepared by milling with anhydrous potassium bromide (KBr) to form very fine powders. Then, the powders were compressed into two thin pellets for analysis. The infrared spectra were recorded with a Fourier transform infrared (FTIR) spectroscopy analyzer (Model JASCO FTIR-6100) within the scanning range 4000–400 cm^−1^. The spectra were smoothed using 3 or 5 points and the baseline of the spectra was corrected using the previously recorded spectra of the sample [60] (Figure 3 and Figure 4).

### 4.6. Antimicrobial Activity of Moringa Leaves

#### 4.6.1. Tested Microorganisms

The inhibitory effect of Moringa ethanolic and aqueous extracts was performed on eight species of phytopathogenic fungi that were used for antifungal testing: 6 serovars of *Aspergillus*, *A. flavus* NRR 3357, *A. ochraceus* ITAL 14, *A. niger* IMI288550, *A. westerdijikia* CCT 6795, *A. carbonarius* ITAL 204, *A. parasiticus* SSWT 2999, *Fusarium proliferatum* MPVP 328, and *Penicillium verrucosum* BFE 500. The stock cultures were grown for 5 days on slant PDA at 25 °C and then kept in a refrigerator until further use. The stock cultures included seven foodborne pathogenic bacterial strains; three Gram-positive bacteria, *Staphylococcus aureus* ATCC 13565, *Staphylococcus sciuri* 2–6, and *Bacillus cereus* EMCC 1080; and four Gram-negative bacteria, *Salmonella enterica* SA19992307, *Salmonella typhi* ATCC 25566, *Escherichia coli* 0157 H7 ATCC 51659, and *Pseudomonas aeruginosa* NRRL B-272. For 24 h, stock cultures were grown on slant nutrient agar at 37 °C and then kept in a refrigerator until further use.

#### 4.6.2. Media Composition

- Tryptic soy broth composition per liter purified water: tryptone (pancreatic digest of casein) 17.0 g, soytone (peptic digest of soybean) 3.0 g, glucose (dextrose) 2.5 g, Sodium Chloride 5.0 g, Dipotassium Hydrogen Phosphate 2.5 g, and pH: 7.3 ± 0.2.
- Potato dextrose agar medium composition: potato infusion 200 g, dextrose20 g, agar20 g, distilled water 1 L, and pH: 7.3 ± 0.2. Note: 200 gm of potato infusion is equivalent to 4.0 g of potato extract.
- YES medium composition: glucose 30 g, yeast extract5 g, Adenine 0.2g, Uracil 0.2 g, Histidine 0.2 g, Leucine 0.2 g, Lysine 0.2 g, agar 20 g, H_2_O to 1 L, and pH: 5.2.

#### 4.6.3. Disc Diffusion Technique

From the 24 h incubated slant nutrient agar of each bacterial species, a loop full of microorganisms was injected into a tube containing 4 to 5 mL of tryptic soy broth (TSB). The broth culture was kept at 35 °C for 2–6 h to obtain the required turbidity of 0.5 McFarland BaSO_4_. A 625 nm spectrophotometer was used to measure the standard turbidity density. With different bacterial cultures, the sensitivity test of the Moringa extracts was determined [61]. Cotton swabs were utilized to create the bacterial cultures from TSB, and Petri dishes were produced with 20 mL of nutrient agar. The discs were positioned on the seeded plates using sterile forceps. Tetracycline (500 g/mL) was utilized as a positive control, and DMSO was used as a negative control. The following day, inoculated plates were incubated at 37 °C for 24 h. At the conclusion of the incubation period, inhibition zones were measured and represented as the diameter of the clear zone including the diameter of the disc.

The fungi strains were plated onto potato dextrose agar (PDA) and cultured at 25 °C for 5 days. “YES” medium Petri dishes were evenly spread with a sterile L-glass rod after being inoculated with 0.05 mL of each fungus culture. The extract-loaded discs were positioned on the seeded plates using sterile forceps. DMSO was utilized as a negative control, while the commercial fungicide Miconazole (1000 unit/mL) was administered as a positive control. The inoculation plates were incubated at 25 °C for 24–48 h. By measuring the inhibition zone (mm) against the tested fungus at the conclusion of the time, the antifungal activity was determined [62]. Three replicas of each treatment were used to calculate the averages of the results of the experiments.

#### 4.6.4. Determination of Minimum Inhibitory Concentration (MIC)

MIC was calculated using the tube dilution technique [63,64]. To achieve 10^8^ CFU mL^−1^ inocula, a 24 h culture of the tested bacterial species was diluted in 10 mL of tryptic soy broth (TSB) according to the 0.5 McFarland standard. Ten different Moringa extract concentrations, ranging from 5000 µg/mL to 10.0 µg/mL, were made in culture tubes with DMSO. Each tube received 0.1 mL of bacterial cell suspension as an inoculant, and it was then incubated for 24 h at 37 °C. Turbidity in the broth served as a sign of inoculum growth, and the extract concentration at which the test organism’s bacterial growth was inhibited at the lowest level was deemed to be the minimal inhibitory concentration (MIC). MIC was carried out against the fungus by the protocol that was designed [65,66]. Different quantities of Moringa extracts were separately diluted in 0.5 mL of 0.1% Tween 80 (Merck, Darmstadt, Germany), combined with 9.5 mL of melting PDA, and then placed into a Petri dish (6 cm). A 3-microliter fungal suspension was used to centrally inoculate the prepared plates (10^8^ CFU mL^−1^, 0.5 McFarland standard). The plates were incubated at 25 °C for 24–48 h.

### 4.7. Statistical Analysis

All tests were run in triplicate for this investigation, and the data were provided as the mean ± SE. For statistical analysis, the “WASP—Web Agri Stat Package- at ICAR: Central Coastal Agricultural Research Institute” was used. The difference between groups was analyzed using a one-way analysis of variation (ANOVA), with the least significant difference (LSD) test, using a 5% threshold of significance (*p* < 0.05) [67].

## 5. Conclusions

It is possible to use Moringa Ag-NPs and their unprocessed aqueous extract to increase antibacterial properties. Because of the nutritious, microbiological, and cell-reinforcing attributes and competencies they demonstrated, the study’s findings suggest that Moringa crude aqueous extract and green chemically synthesized Ag-NPs from it can be developed into various promising food and biomedical applications. However, this suggestion must take into account the significant cytotoxic activity displayed by synthesized Ag-NPs and the crude extracts at elevated concentrations.

## Figures and Tables

**Table 1:** Chemical composition of Moringa leaves.

Parameter	Conc. (%)
Moisture	7.94 ± 0.10
DM	92.06 ± 0.10
OM	88.35 ± 0.54
Ash	11.65 ± 0.54
Total Lipid	13.55 ± 1.10
TP Crude	28.59 ± 0.11
CF	32.15 ± 1.87
NFE	14.05 ± 2.68

**Table 2:** Mineral composition of Moringa leaves.

Element	Conc. (ppm)
Mg	147.5 ± 10.5
Ca	1110 ± 7.00
K	559.00 ± 14.05
Na	21.50 ± 1.50
Zn	0.125 ± 0.05
Cu	0.53 ± 0.09

**Table 3:** The total amount of phenolic and flavonoid compounds of different successive extracts of Moringa.

Extracts	Total Phenols/Flavonoids (µg/g) * (Mean ± SE)
TPCs	
Hexane	22.93 ± 0.12 ^c^
Petroleum ether	12.72 ± 0.13 ^d^
Ethyl acetate	32.96 ± 0.58 ^a^
Ethanol	30.94 ± 0.57 ^b^
Aqueous	32.66 ± 0.21 ^a^
Treatments were found significant at a 5% level of significance and LSD (0.05) = 1.211	
TFCs	
Hexane	231.40 ± 5.29 ^d^
Petroleum ether	117.40 ± 5.03 ^e^
Ethyl acetate	462.73 ± 1.76 ^c^
Ethanol	540.07 ± 12.98 ^b^
Aqueous	804.73 ± 12.02 ^a^
Treatments were found significant at a 5% level of significance and LSD (0.05) = 27.080	

* µg/g plant extract as gallic acid or Quercetin equivalents. Values with the same letter are not significantly different at (*p* ≤ 0.05).

**Table 4:** Antibacterial activity of Moringa ethanolic and aqueous extracts against different bacterial strains by disc diffusion method.

	Inhibition Zone (mm) (Mean ± SE)			
Pathogenic Bacterial Strains	−Ve Ctrl	+Ve Ctrl	Ethanol	Aqueous
*Bacillus cereus*	0.00	26.33 ± 0.72 ^ab^	0.00 ^ns^	7.66 ± 0.16 ^b^
*Staphylococcus sciuri*	0.00	28.83 ± 0.44 ^a^	7.33 ± 0.33 ^c^	7.83 ± 0.16 ^b^
*Staphylococcus aureus*	0.00	26.00 ± 1.25 ^ab^	7.33 ± 0.16 ^c^	8.16 ± 0.16 ^b^
*Escherichia coli*	0.00	10.83 ± 0.72 ^d^	8.00 ± 0.00 ^bc^	10.00 ± 0.29 ^a^
*Salmonella typhi*	0.00	26.00 ± 1.25 ^ab^	9.33 ± 0.17 ^b^	10.33 ± 0.60 ^a^
*Salmonella enterica*	0.00	23.16 ± 1.01 ^b^	10.83 ± 1.01 ^a^	10.16 ± 0.33 ^a^
*Pseudomonas aeruginosa*	0.00	17.00 ± 1.61 ^c^	8.17 ± 0.44 ^bc^	10.67 ± 0.60 ^a^
LSD _(0.01)_	0.00	4.509	1.875	1.591
LSD _(0.05)_	0.00	3.249	1.351	1.147
Coefficient of variation	0.00	8.209	10.589	7.068

Each value represents the mean ± SE. The same letter over values denotes that they are not significantly different at (*p* ≤ 0.05), and comparison is conducted according to each extract with all strains. +Ve Ctrl is Tetracycline and −Ve Ctrl is DMSO.

**Table 5:** Antifungal activity of Moringa ethanolic and aqueous extracts against different fungal strains by disc diffusion method.

	Inhibition Zone (mm) (Mean ± SE)			
Phytopathogenic Fungal Strains	−Ve Ctrl	+Ve Ctrl	Ethanol	Aqueous
*Aspergillus flavus*	0.00	21.33 ± 1.01 ^bc^	8.00 ± 0.58 ^a^	8.50 ± 0.76 ^c^
*Aspergillus parasiticus*	0.00	22.33 ± 1.01 ^ab^	8.83 ± 0.83 ^a^	10.33 ± 0.60 ^bc^
*Aspergillus niger*	0.00	23.83 ± 0.73 ^a^	7.50 ± 0.50 ^a^	12.67 ± 0.73 ^a^
*Aspergillus carbonarius*	0.00	17.83 ± 0.73 ^cd^	8.33 ± 0.73 ^a^	10.50 ± 0.29 ^bc^
*Aspergillus ochraceus*	0.00	20.83 ± 0.73 ^bc^	8.83 ± 0.17 ^a^	10.83 ± 0.93 ^ab^
*Aspergillus westerdijikia*	0.00	19.50 ± 0.76 ^cd^	8.17 ± 0.44 ^a^	9.50 ± 0.29 ^bc^
*Fusarium proleferatum*	0.00	16.50 ± 0.87 ^e^	8.33 ± 0.33 ^a^	10.00 ± 0.29 ^bc^
*Penicillium verrucosum*	0.00	22.83 ± 0.44 ^ab^	8.33 ± 0.60 ^a^	11.00 ± 1.04 ^ab^
LSD _(0.01)_	0.00	3.320	Nill	Nill
LSD _(0.05)_	0.00	2.409	Nill	2.030
Coefficient of variation	0.00	6.749	11.677	11.257

Each value represents the mean ± SE. The same letter over values denotes that they are not significantly different at (*p* ≤ 0.05), and comparison is performed according to each extract with all strains. +Ve Ctrl is Miconazole and −Ve Ctrl is DMSO.

**Table 6:** The minimum inhibitory concentration of different Moringa extracts against bacterial strains.

Moringa Ethanolic and Aqueous Extracts and Bacterial Strains MIC (mg/mL) (Mean ± SE)		
Pathogenic Bacterial Strains	Ethanol	Aqueous
*Bacillus cereus*	0.83 ± 0.33 ^c^	0.33 ± 0.08 ^a^
*Staphylococcus sciuri*	4.17 ± 0.83 ^a^	0.27 ± 0.13 ^a^
*Staphylococcus aureus*	2.67 ± 1.17 ^ab^	0.03 ± 0.01 ^a^
*Escherichia coli*	0.18 ± 0.07 ^c^	0.04 ± 0.01 ^a^
*Salmonella typhi*	0.42 ± 0.08 ^c^	0.27 ± 0.13 ^a^
*Salmonella enterica*	0.67 ± 0.17 ^c^	0.33 ± 0.08 ^a^
*Pseudomonas aeruginosa*	1.33 ± 0.17 ^bc^	0.33 ± 0.08 ^a^
LSD _(0.01)_	2.378	Nill
LSD _(0.05)_	1.714	Nill
Coefficient of variation	66.709	66.982

Each value represents the mean ± SE. The same letter over values denotes that they are not significantly different at (*p* ≤ 0.05), and comparison is conducted according to each extract with all strains.

**Table 7:** The minimum inhibitory concentration of different Moringa extracts against fungal strains.

Moringa Ethanolic and Aqueous Extracts and Fungal Strains MIC (mg/mL) (Mean ± SE)		
Phytopathogenic Fungal Strains	Ethanol	Aqueous
*Aspergillus flavus*	0.04 ± 0.01 ^b^	0.67 ± 0.17 ^a^
*Aspergillus parasiticus*	0.04 ± 0.01 ^b^	0.42 ± 0.08 ^a^
*Aspergillus niger*	0.12 ± 0.07 ^b^	1.17 ± 0.33 ^a^
*Aspergillus carbonarius*	0.42 ± 0.08 ^a^	1.17 ± 0.33 ^a^
*Aspergillus ochraceus*	0.20 ± 0.15 ^ab^	1.17 ± 0.17 ^a^
*Aspergillus westerdijikia*	0.04 ± 0.01 ^b^	1.17 ± 0.33 ^a^
*Fusarium proleferatum*	0.42 ± 0.08 ^a^	0.83 ± 0.17 ^a^
*Penicillium verrucosum*	0.18 ± 0.07 ^b^	1.17 ± 0.17 ^a^
LSD _(0.01)_	0.312	Nill
LSD _(0.05)_	0.226	Nill
Coefficient of variation	71.964	40.994

Each value represents the mean ± SE. The same letter over values denotes that they are not significantly different at (*p* ≤ 0.05), and comparison is performed according to each extract with all strains.

**Table 8:** Antibacterial activity of Moringa crude aqueous extracts and synthesized Ag-NPs against different bacterial strains by disc diffusion method.

Inhibition Zone (mm) (Mean ± SE)			
Pathogenic Bacterial Strains	+Ve Control	Moringa Crude	Ag-NPs from Moringa
*Bacillus cereus*	10.00 ± 0.29 ^d^	7.50 ± 0.29 ^bc^	9.17 ± 0.44 ^a^
*Staphylococcus sciuri*	26.83 ± 0.60 ^a^	7.67 ± 0.33 ^bc^	9.50 ± 0.58 ^a^
*Staphylococcus aureus*	16.00 ± 0.87 ^c^	8.17 ± 0.17 ^ab^	9.33 ± 0.17 ^a^
*Escherichia coli*	10.17 ± 0.33 ^d^	7.00 ± 0.00 ^c^	9.33 ± 0.17 ^a^
*Salmonella typhi*	27.33 ± 0.44 ^a^	7.50 ± 0.29 ^bc^	10.67 ± 0.44 ^a^
*Salmonella enterica*	24.00 ± 0.87 ^b^	8.00 ± 0.29 ^ab^	9.50 ± 0.58 ^a^
*Pseudomonas aeruginosa*	16.83 ± 0.60 ^c^	8.83 ± 0.44 ^a^	10.67 ± 0.17 ^a^
LSD _(0.01)_	2.571	Nill	Nill
LSD _(0.05)_	1.853	0.876	Nill
Coefficient of variation	5.645	6.402	7.174

Each value represents the mean ± SE. The same letter over values denotes that they are not significantly different at (*p* ≤ 0.05), and comparison is conducted according to each sample with all strains. +Ve Ctrl is Tetracycline and −Ve Ctrl is DMSO.

**Table 9:** Antifungal activity of Moringa crude aqueous extracts and synthesized Ag-NPs against different fungal strains by disc diffusion method.

Inhibition Zone (mm) (Mean ± SE)			
Phytopathogenic Fungal Strains	+Ve Control	Moringa Crude	Ag-NPs from Moringa
*Aspergillus flavus*	20.50 ± 1.00 ^a^	8.33 ± 0.60 ^a^	9.83 ± 0.88 ^a^
*Aspergillus parasiticus*	21.83 ± 0.67 ^a^	7.83 ± 0.33 ^a^	9.00 ± 0.00 ^a^
*Aspergillus niger*	21.33 ± 0.44 ^a^	8.67 ± 0.17 ^a^	10.50 ± 0.29 ^a^
*Aspergillus carbonarius*	21.50 ± 0.76 ^a^	7.83 ± 0.17 ^a^	9.83 ± 0.88 ^a^
*Aspergillus ochraceus*	17.50 ± 1.00 ^b^	7.83 ± 0.44 ^a^	9.17 ± 0.44 ^a^
*Aspergillus westerdijikia*	20.50 ± 1.04 ^a^	8.33 ± 0.60 ^a^	9.83 ± 0.60 ^a^
*Fusarium proleferatum*	15.83 ± 0.67 ^b^	8.17 ± 0.44 ^a^	10.17 ± 0.44 ^a^
*Penicillium verrucosum*	15.25 ± 1.25 ^b^	8.17 ± 0.17 ^a^	10.33 ± 0.44 ^a^
LSD _(0.01)_	3.245	Nill	Nill
LSD _(0.05)_	2.355	Nill	Nill
Coefficient of variation	7.042	8.568	10.009

Each value represents the mean ± SE. The same letter over values denotes that they are not significantly different at (*p* ≤ 0.05), and comparison is performed according to each sample with all strains. +Ve Ctrl is Miconazole and −Ve Ctrl is DMSO.

**Table 10:** The minimum inhibitory concentration of Moringa crude aqueous extracts and synthesized Ag-NPs against different bacterial strains.

MIC (mg/mL) (Mean ± SE)		
Pathogenic Bacterial Strains	Moringa Crude	Ag-NPs from Moringa
*Bacillus cereus*	0.08 ± 0.02 ^b^	0.07 ± 0.02 ^a^
*Staphylococcus sciuri*	0.28 ± 0.12 ^b^	0.13 ± 0.06 ^a^
*Staphylococcus aureus*	0.83 ± 0.08 ^a^	0.05 ± 0.00 ^a^
*Escherichia coli*	0.15 ± 0.05 ^b^	0.05 ± 0.00 ^a^
*Salmonella typhi*	0.67 ± 0.08 ^a^	0.05 ± 0.00 ^a^
*Salmonella enterica*	0.12 ± 0.07 ^b^	0.05 ± 0.00 ^a^
*Pseudomonas aeruginosa*	0.67 ± 0.17 ^a^	0.07 ± 0.02 ^a^
LSD _(0.01)_	0.398	Nill
LSD _(0.05)_	0.287	Nill
Coefficient of variation	40.916	63.387

Each value represents the mean ± SE. The same letter over values denotes that they are not significantly different at (*p* ≤ 0.05), and comparison is conducted according to each sample with all strains.

**Table 11:** The minimum inhibitory concentration of Moringa crude aqueous extracts and synthesized Ag-NPs against different fungal strains.

MIC (mg/mL) (Mean ± SE)		
Phytopathogenic Fungal Strains	Moringa Crude	Ag-NPs from Moringa
*Aspergillus flavus*	1.08 ± 0.17 ^b^	0.83 ± 0.08 ^a^
*Aspergillus parasiticus*	1.17 ± 0.08 ^b^	0.83 ± 0.08 ^a^
*Aspergillus niger*	0.92 ± 0.08 ^b^	0.28 ± 0.12 ^abc^
*Aspergillus carbonarius*	1.08 ± 0.08 ^b^	0.42 ± 0.08 ^bc^
*Aspergillus ochraceus*	1.00 ± 0.14 ^b^	0.33 ± 0.08 ^bc^
*Aspergillus westerdijikia*	0.83 ± 0.08 ^b^	0.25 ± 0.00 ^c^
*Fusarium proleferatum*	0.74 ± 0.14 ^b^	0.67 ± 0.08 ^ab^
*Penicillium verrucosum*	3.33 ± 0.83 ^a^	0.83 ± 0.17 ^a^
LSD _(0.01)_	1.300	0.487
LSD _(0.05)_	0.943	0.353
Coefficient of variation	42.919	34.379

Each value represents the mean ± SE. The same letter over values denotes that they are not significantly different at (*p* ≤ 0.05), and comparison is performed according to each sample with all strains.

## Data Availability

All data are available within the manuscript.
